# Comparison of Abiraterone and Combined Androgen Blockade Therapy for High-Risk Metastatic Hormone-Sensitive Prostate Cancer: A Propensity Score-Matched Analysis

**DOI:** 10.3389/fonc.2021.769068

**Published:** 2021-12-21

**Authors:** Naoki Matsumura, Kazutoshi Fujita, Mitsuhisa Nishimoto, Yutaka Yamamoto, Ken Kuwahara, Yasuharu Nagai, Takafumi Minami, Yuji Hatanaka, Masahiro Nozawa, Yasuhiro Morimoto, Hideo Tahara, Shigeya Uejima, Atsunobu Esa, Akihide Hirayama, Kazuhiro Yoshimura, Hirotsugu Uemura

**Affiliations:** ^1^ Department of Urology, Mimihara General Hospital, Sakai Sakai-ku, Japan; ^2^ Department of Urology, Faculty of Medicine, Kindai University Hospital, Osakasayama, Japan; ^3^ Department of Urology, Faculty of Medicine, Kindai University Nara Hospital, Ikoma, Japan; ^4^ Department of Urology, Kaizuka City Hospital, Kaizuka, Japan; ^5^ Department of Urology, National Hospital Organization Osaka Minami Medical Center, Kawachinagano, Japan; ^6^ Department of Urology, Saiseikai Tondabayashi Hospital, Tondabayashi, Japan; ^7^ Department of Urology, Morimoto Urology Clinic, Sakai Minami-ku, Japan

**Keywords:** prostate cancer, metastatic hormone-sensitive prostate cancer, abiraterone acetate, combined androgen blockade, high risk prostate cancer

## Abstract

This study aimed to compare the effects of abiraterone acetate plus prednisone (AAP) with androgen deprivation therapy (ADT) with those of combined androgen blockade (CAB) therapy in patients with high-risk metastatic hormone-sensitive prostate cancer (mHSPC). This study retrospectively identified 163 patients with high-risk mHSPC at Kindai University and affiliated hospitals between January 2014 and December 2020. Kaplan-Meier analysis was used to summarize progression-free survival (PFS) and overall survival (OS). Multivariate Cox proportional hazard modeling was used to identify the prognostic factors in the overall cohort. Propensity score matching was used to adjust the clinical characteristics, and log-rank test was applied to these propensity score–matched cohorts. Seventy-four patients who received AAP with ADT and 89 patients who received CAB were included in this study. The median follow-up duration was 27 months (range, 2–89 months). The median PFS and OS were not reached by the AAP+ADT group and 15 and 79 months, respectively, in the CAB group. The Eastern Cooperative Oncology Group (ECOG) performance status (PS) score and AAP+ADT were significant prognostic factors for PFS, whereas ECOG PS score, visceral metastasis, and AAP+ADT were significant prognostic factors for OS. The 2-year PFS was 76.1% in the AAP+ADT group and 38.6% in the CAB group (P < 0.0001), and the 2-year OS was 90.2% in the AAP+ADT group and 84.8% in the CAB group (P = 0.015). In conclusion, AAP+ADT had better PFS and OS than CAB in patients with high-risk mHSPC.

## Introduction

Prostate cancer is the most common cancer and the second most common cause of death in the United States. The 5-year relative survival rate of patients with metastatic prostate cancer is 30% (1). The U.S. Preventive Services Task Force recommends individualized decision making, not blanked against prostate-specific antigen (PSA) screening for prostate cancer, but the incidence of metastatic prostate cancer has been increasing rapidly since 2012 (2). Metastatic hormone-sensitive prostate cancer (mHSPC) accounts for up to 5% of patients newly diagnosed with prostate cancer in the United States (3). Androgen deprivation therapy (ADT) with a luteinizing hormone-releasing hormone agonist or receptor antagonist or with bilateral orchiectomy has been the standard of care for men with mHSPC. Combined androgen blockade (CAB) therapy with a standard nonsteroidal antiandrogen drug (bicalutamide or flutamide) is not recommended with systemic therapy for castration-naïve disease according to the National Comprehensive Cancer Network clinical practice guidelines in oncology, but it is recommended as grade B in the Japanese Urological Association guidelines. CAB therapy may be superior to other hormone therapies for overall survival (OS) in patients with mHSPC in Japan (4). The rate of CAB therapy in primary hormone therapy is higher in Japan than in Western Europe, and the cancer-specific mortality rate is less than half that in the United States (5). The dosage of bicalutamide in Japan and Western Europe differs (80 and 50 mg, respectively).

In the LATITUDE trial, addition of abiraterone acetate plus prednisone (AAP) to ADT significantly prolonged progression-free survival (PFS) and OS compared with ADT in high-risk mHSPC, who were defined as having at least two of the following three high-risk factors: Gleason score ≥8, visceral metastasis, and ≥3 bone metastases (6). In the ENZAMET trial, addition of enzalutamide to ADT resulted in longer PFS and OS within 3 years than CAB therapy (7). However, whether the addition of AAP to ADT improves PFS and OS in patients with high-risk mHSPC compared with CAB therapy remains unknown. The aim of our study was to compare the effect of AAP+ADT with CAB therapy for upfront treatment in patients with high-risk mHSPC.

## Materials and Methods

This study retrospectively identified 166 patients with high-risk mHSPC at Kindai University and its affiliated hospitals from January 2014 to December 2020. All patients were adult men with pathologically diagnosed prostate adenocarcinomas and had not received prior hormonal therapy. Patients with high-risk HSPC were defined as having at least two of the following three high risk factors: Gleason score ≥8, visceral metastasis, and ≥3 bone metastases. Three patients with no prostate biopsy or unknown prognosis were excluded. The data of 163 patients were retrospectively analyzed. The metastasis burden was evaluated using computed tomography and diffusion-weighted whole-body imaging with background body signal or bone scan. Eligible patients had an Eastern Cooperative Oncology Group (ECOG) performance status (PS) score <3. Disease progression was assessed using the Prostate Cancer Working Group 2. PSA progression was defined as a confirmed relative increase in the PSA level from the nadir value by ≥25% and ≥2 ng/ml. The extent of disease (EOD) score was used to classify bone metastases. This study was approved by the institutional ethics committee of Kindai University (R02-247), and written informed consent was waived owing to the retrospective design.

### Statistical Analysis

Clinical characteristics were analyzed using the Mann-Whitney U test and Fisher’s exact test. Kaplan-Meier analysis was used to summarize PFS and OS. Differences in time events were compared using log-rank tests. Hazard ratios (HRs) and their 95% confidence intervals (CIs) were estimated using Cox proportional hazards regression to investigate the factors affecting PFS and OS. Variables included age, PSA, Gleason score, ECOG PS score, visceral metastasis, and AAP+ADT. Propensity score matching was used to adjust patient characteristics to a 1:1 ratio between the AAP+ADT and CAB groups. The propensity score was calculated using logistic regression models with age, PSA, Gleason score, ECOG PS score, visceral metastasis, and EOD score. The propensity scores were estimates of the probability of receiving AAP. With propensity score matching, one produces individual pairs of patients, one from each treatment, that were matched on an individual basis. A matched analysis that takes 105 individual pairing into account was carried out. Probability values (P) and CIs were two-sided, and a P value <0.05 was considered significant. All statistical analyses were performed with EZR (Saitama Medical Center, Jichi Medical University, Saitama, Japan), which is a modified version of the R packages designed to add statistical functions frequently used in biostatistics.

## Results

### Patients, Treatment, and Safety

Among 163 patients with high-risk mHSPC, 74 patients (45.4%) received AAP (abiraterone acetate 1000 mg+ prednisone 5 mg daily) +ADT and 89 patients (54.6%) received CAB (bicalutamide 80 mg daily). The baseline characteristics are summarized in **Table 1**. The median follow-up duration was 27 months (range, 2–89 months). There was no significant difference in clinical characteristics between the two groups. Sixteen (21.6%) of 74 patients in the AAP+ADT group and 65 (73.0%) of 89 patients in the CAB group progressed to castration-resistant prostate cancer (CRPC). The secondary therapies were enzalutamide (58.3%) and docetaxel (25.0%) in the AAP+ADT group and flutamide (41.0%), abiraterone (19.7%), and docetaxel (18.0%) in the CAB group (**Supplementary Table 1**). The most frequently used subsequent therapy was enzalutamide (72.7%) in the AAP+ADT group and abiraterone (50.8%) in the CAB group (**Table 2**). Eight (72.7%) of 11 CPRC patients in the AAP+ADT group received next-generation androgen receptor signaling inhibitor (ARSI) as subsequent therapy, whereas 45 (69.5%) of 61 CRPC patients in the CAB group received next-generation ARSI. There was no significant difference between the two groups regarding the use of the next-generation ARSI and taxane for CPRC (P = 1 and P = 0.31, respectively, Fisher’s exact test). Treatment-emergent adverse events leading to treatment discontinuation were reported in 5 (6.8%) of 74 patients in the AAP+ADT group and in 1 (1.1%) of 89 patients in the CAB group. The details of serious adverse events are summarized in **Table 3**. There was no significant difference between 2 groups regarding the serious adverse events (Fisher’s exact test, P=0.09). No treatment-emergent adverse events that led to death were reported in either group. No patient died within 30 days of prostate cancer treatment.

**Table 1:** Characteristics of the patients at baseline.

Characteristic	AAP+ADT (n = 74)	CAB (n = 89)	P-value
Age (years), median (range)	74 (53-88)	74 (52-88)	0.54
**PSA (ng/ml), median (range)**	456 (4.6-11,507)	241 (8.8-11371)	0.279
ECOG PS score (no, %)			
0	46 (62.2)	54 (60.7)	0.873
≥1	28 (37.8)	35 (39.3)	
Gleason score (no, %)			
<8	0 (0)	1 (1.1)	1
≥8	74 (100)	88 (98.9)	
Metastasis site			
Lymph node	29 (39.2)	31 (34.8)	0.626
Bone	71 (96.0)	86 (96.6)	1
Visceral	20 (27.0)	20 (22.5)	0.584
EOD			
0	4 (5.4)	3 (3.4)	0.063
1	26 (35.1)	18 (20.2)	
≥2	44 (59.5)	68 (76.4)	
**ALP (lU/ml), median** (range)	395 (84-4,797)	431 (70-24,280)	0.794
**LDH (lU/ml), median** (range)	208 (137-4,220)	204 (135-1,334)	0.252

AAP, abiraterone acetate plus prednisone; ADT, androgen deprivation therapy; CAB, combined androgen blockade; PSA, prostate-specific antigen; ECOG PS, Eastern Cooperative Oncology Group performance status; EOD, extent of disease ; ALP, alkaline phosphatase; LDH, lactose dehydrogenase.

**Table 2:** Subsequent therapy for mHSPC patients who have progressedto mCRPC.

Summary of subsequent therapy, n (%)	AAP+ADT (n = 11)	CAB (n = 61)
		
**Flutamide**	0 (0)	23 (37.7)
Enzalutamide	8 (72.7)	19 (31.1)
Abiraterone	0 (0)	31 (50.8)
Apalutamide	1 (9.1)	2 (3.3)
Darolutamide	0 (0)	2 (3.3)
Docetaxei	6 (54.5)	21 (34.4)
Cabazitaxel	1 (9.1)	9 (14.8)
**Radium-223 chloride**	0 (0)	2 (3.3)
Dexamethasone	1 (9.1)	5 (8.2)
Etinolestridol	0 (0)	6 (9.8)

mHSPC, metastatic hormone-sensitive prostate cancer; mCRPC,metastatic castration-resistant prostate cancer; AAP, abiraterone acetate plus prednisone; ADT, androgen deprivation therapy; CAB, combined androgen blockade.

**Table 3:** Treatment-emergent adverse eventsleading to treatment discontinuation.

Summary of TEAEs, n (%)	AAP+ADT (n = 74)	CAB (n = 8B)
		
AE leading to death	0	0
AE leading to treatment discontinuation	5 (6.8]	1 (1.1)
Grade 3 events		
Vertigo	1 (14]	0
Fatigue	1 (1.4)	0
Hypokalemia	2 (2.8)	0
ALT increased	1 (14]	1 (1.1)
AST increased	1 (14]	1 (1.1)
ALP increased	1 (14]	0

TEAEs. Treatment-emergent adverse events; AAP, abiraterone acetate plus prednisone, ADT. androgen deprivation therapy; CAB, combined androgen blockade; AE, adverse event; ALT, alanine aminotransferase; AST, aspartate aminotransferase: ALP, alkaline phosphatase.

### Progression-Free and Overall Survival

**Figure 1:**
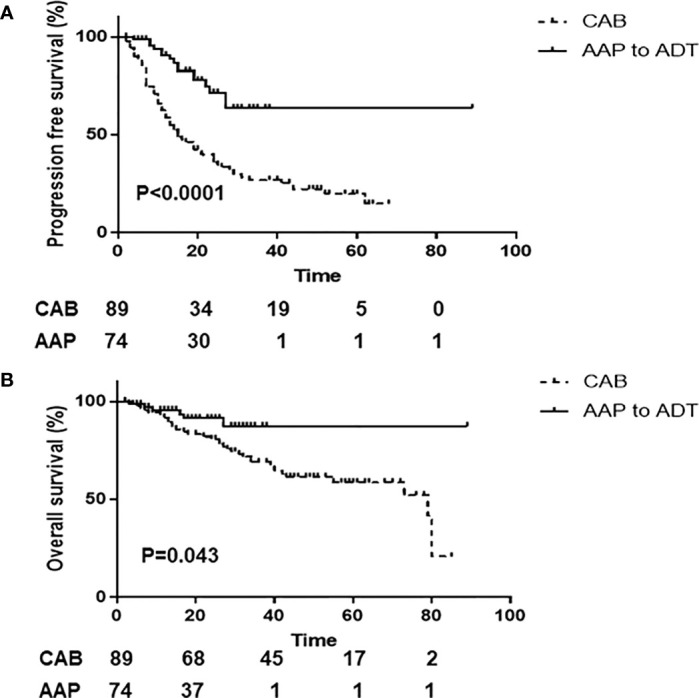
Kaplan-Meier analysis of men with high-risk metastatic hormone-sensitive prostate cancer stratified by AAP+ADT and CAB therapy. Progression-free survival **(A)** and overall survival **(B)** in the overall population. AAP+ADT, addition of abiraterone acetate plus prednisone to androgen deprivation therapy; CAB, combined androgen blockade.

At the time of the analysis, 40 patients died. The median PFS and OS were not reached in the AAP+ADT group, but they were 15 and 79 months, respectively, in the CAB group. The 2-year PFS was 71.5% (95% CI, 55.2%) in the AAP+ADT group and 36.1% (95% CI, 25.8%–46.4%) in the CAB group, respectively (P < 0.0001; **Figure 1A**). The 2-year OS was 91.3% (95% CI, 80.1%–96.4%) in the AAP+ADT group and 80.8% (95% CI, 80.4%–90.4%) in the CAB group (P = 0.043; **Figure 1B**). In the univariate Cox proportional hazards analysis, the ECOG PS score and AAP+ADT were significantly associated with PFS. Multivariate analysis showed that ECOG PS score and AAP+ADT were significantly associated with PFS (**Table 4**). In the univariate Cox proportional hazards analysis, age, ECOG PS score, and AAP+ADT were significantly associated with OS. The multivariate analysis for the prediction of overall survival showed that the ECOG PS score, presence of visceral metastasis, and AAP+ADT were associated with OS (**Table 5**).

**Table 4:** Cox regression analysis of progression free survival.

Variable	Univariate analysis			Multivariateanalysis		
	HR	95% Cl	P-value	HR	95% Cl	P-value
Age	1.05	0.99-1.09	0.056	1.04	0.99-1.09	0.096
**PSA**	0.99	0.99-1.00	0.312	0.99	0.99-1.00	0.264
**Gleasonscore(≥8vs6-7)**			0.997			0.996
**ECOG PS score (1-3 vs O)**	2.55	1.36-4.76	0.003	3.02	1.59-5.75	<0.001
**Visceral metastasis (yes vs no)**	1.53	0.77-3.00	0.222	2.00	0.99-4.02	0.051
**AAP+ADT (yes vs no)**	0.20	0.08-0.48	<0.001	0.09	0.07-0.40	<0.001

AAP, abiraterone acetate plus prednisone; ADT, androgen deprivation therapy; PSA, prostate-specific antigen; ECOG PS, Eastern Cooperative Oncology Group performance status; HR, hazards ratio; Cl, confidence interval.

**Table 5:** Cox regression analysis of overall survival.

Variable	Univariate analysis			Multivariate analysis		
	HR	95% Cl	P-value	HR	95% Cl	P-value
Age	1.05	1.00-1.11	0.042	1.05	0.99-1.11	0.069
**PSA**	0.99	0.99-1.00	0.408	0.99	0.99-1.00	0.285
**Gleasanscore (≥8 vs6-7)**			0.997			0.998
**ECOG PS score (1 -3 vs 0)**	2.77	1.47-5.21	0.002	2.94	1.53-5.64	0.001
**Visceral metastasis (yes vs no)**	1.85	0.94-3.67	0.077	2.49	1.21-5.09	0.013
**AAP+ADT (yes vs no)**	0.41	0.17-0,99	0.049	0.36	0.15-0.91	0.031

AAP, abiraterone acetate plus prednisone; ADT, androgen deprivation therapy; PSA, prostate-specific antigen; ECOG PS, Eastern Cooperative Oncology Group performance status; HR, hazards ratio; Cl, confidence interval.

### Propensity Score–Matched Analysis

**Figure 2:**
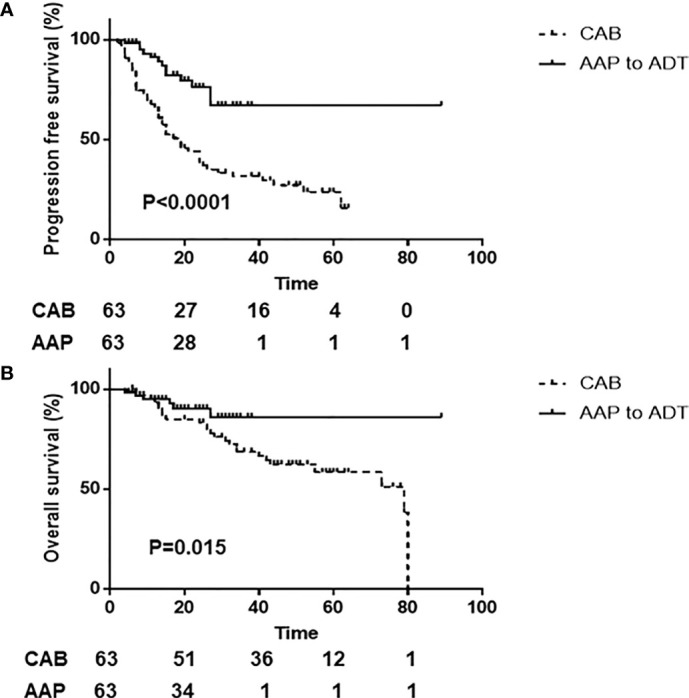
Kaplan-Meier analysis of men with high-risk metastatic hormone-sensitive prostate cancer stratified by AAP+ADT and CAB therapy. Progression-free survival **(A)** and overall survival **(B)** in the propensity score–matched cohorts. AAP+ADT, addition of abiraterone acetate plus prednisone to androgen deprivation therapy; CAB, combined androgen blockade.

Propensity score matching was used because a selection bias for the use of AAP could exist, resulting in matched cohorts of 63 patients with AAP+ADT and 63 patients with CAB. The clinical characteristics after matching are summarized in **Table 6**. The clinical characteristics were well adjusted between the two groups. The 2-year PFS was 76.1% (95% CI, 60.3%) in the AAP+ADT group and 38.6% (95% CI, 26.7%–74.7%) in the CAB group (P < 0.0001; **Figure 2A**). The 2-year OS was 90.2% (95% CI, 77.9%–90.9%) in the AAP +ADT group and 84.8% (95% CI, 72.8%–90.8%) in the CAB group (P = 0.015; **Figure 2B**). AAP+ADT significantly improved OS and PFS compared with CAB, even in the propensity score–matched cohorts of patients with high-risk mHSPC.

**Table 6:** Characteristicsofthe propensity scorematched patients at baseline.

Characteristic	AAP+ADT (n=63)	CAB (n=63)	P-value
Age (years), median (range)	75 (58-88)	73 (62-84)	0.40
PSA (ng/ml), median (range)	450 (4.6-11,507)	232 (11.0-11371)	0.373
ECOG PS score (no, %)			
0	41 (65.1)	42 (66.7)	1
≥1	22 (34.9)	21 (33.3)	
Gleason score (no, %)			
<8	0 (0)	0 (0)	1
≥8	63 (100|	63 (100|	
Metastasis site			
Lymph node	22 |34.9|	25 (39.7)	0.713
Bone	60 (95.2)	61 (96.8)	1
Visceral	18 (28.6)	17 (27.0)	1
EOD			
0	3 (4.8)	3 (4.8)	0.950
1	19 (30.1)	17 (26.9)	
*≥2*	41 (65.1)	43 (68.3)	
ALP (lU/ml), median (range)	402 (84-4,797)	377 (70-24,280)	0.S88
LDH (IU/ml), median (range)	208 (137-4,220)	214 (136-729)	0.246

AAP, abirateroiie acetate plus prednisone; ADT, androgen deprivation therapy; CAR. combined androgen blockade; PSA, pro state^-^specific antigen; ECOG PS_r_ Eastern Cooperative Oncology Group performance status, EOD. extent of disease’, ALP, alkaline phosphatase; LDH, lactose dehydrogenase.

## Discussion

This study retrospectively analyzed the efficacy of AAP+ADT in patients with high-risk mHSPC compared with CAB therapy and found that AAP+ADT significantly improved PFS and OS by propensity score–matched analysis. In the CHAARTED and STAMPEDE (arm C), addition of docetaxel to ADT resulted in beneficial effects on PFS and OS in patients with mHSPC (8, 9). In the LATITUDE and STAMPEDE (arm G) trial, AAP+ADT resulted in beneficial effects on PFS and OS in patients with high-risk mHSPC (6, 10). In the ENZAMET trial, addition of enzalutamide to CAB therapy resulted in beneficial effects on PFS and OS in patients with mHSPC (7). In the ARCHES trial, addition of enzalutamide to ADT resulted in a reduced risk of metastatic progression or death in patients with mHSPC, including after docetaxel chemotherapy (11). In the TITAN trial, addition of apalutamide to ADT resulted in beneficial effects on radiographic PFS and OS in patients with mHSPC (12). Based on these studies, docetaxel, AAP, enzalutamide, and apalutamide are recommended for patients with mHSPC. In the Japanese subgroup analyses of LATITUDE with high-risk mHSPC patients, a reduced risk of radiographic PFS and PSA-PFS in Japanese patients was observed in the AAP group compared with the ADT group and the AAP+ADT group had a favorable treatment effect on OS than the ADT group (13, 14).

Results from LATITUDE and STAMPEDE (arm G) showed that Gleason score, ECOG PS score, and nodal status were not prognostic factors for OS in mHSPC patients treated with AAP+ADT compared with those treated with ADT alone, but the benefit of AAP+ADT was greater in younger men (15). Miyazawa et al. (2021) reported that the prognostic factors for OS in high-risk mHSPC patients treated with CAB therapy were ECOG PS score, hemoglobin, and PSA response after 3 months (<97.0%) (16).

In our study, PFS and OS were significantly longer in the AAP+ADT group than in the CAB group. Ueda et al. retrospectively compared 50 high-risk mHSPC patients with AAP+ADT and 99 patients with CAB therapy, and showed the benefit of AAP+ADT for improvement of overall survival in Japanese patients with high-risk mHPSC (17). However, the sample size was small and further accumulation of evidence is necessary. In their study, LHRH antagonist were used as ADT, while LHRH agonists were used in our study. Our results confirmed the efficacy of AAP+ADT in 74 patients compared with CAB. While Ueda et al. reported that the prognostic factor for PSA-PFS in high-risk mHSPC patients was a high Gleason score, the ECOG PS score and visceral metastasis were poor prognostic factors for OS in our study. Docetaxel may be an alternative option for patients with these factors.

Since 2014, ARSI has been used for CPRC treatment in Japan, and the prognosis of patients with high-risk mHSPC treated with CAB has been prolonged. Our study included cases from 2014, who have received ARSI and taxane after the diagnosis of CRPC. Secondary therapy after progression to CRPC includes enzalutamide and docetaxel in the AAP+ADT group and flutamide, enzalutamide, and docetaxel in the CAB group. The subsequent therapies were docetaxel and ARSI in both groups. Bicalutamide did not improve the OS of patients with high-risk HSPC, regardless of whether most of the patients in the CAB group received ARSI (enzalutamide or abiraterone) after the diagnosis of CRPC. Our results suggest that the upfront use of AAP instead of bicalutamide followed by ARSI resulted in prolonged OS in patients with high-risk mHSPC.

The present study has several limitations. This was retrospective study with a small cohort and a short observation period. Further larger-scale studies should be performed to compare the effect of AAP+ADT with CAB therapy for upfront treatment in patients with high-risk mHSPC.

## Conclusion

In this study, AAP+ADT provided better PFS and OS than CAB therapy in patients with high-risk mHSPC. Upfront AAP+ADT would be recommended for patients with high-risk mHSPC.

## Data Availability Statement

The original contributions presented in the study are included in the article/**Supplementary Material**. Further inquiries can be directed to the corresponding author.

## Ethics Statement

The studies involving human participants were reviewed and approved by the institutional ethics committee of Kindai University (R02-247). The patients/participants provided their written informed consent to participate in this study.

## Author Contributions

NM and KF performed concept and design of experiments. Acquisition of data was done by NM, MNi, YY, KK, YH, YM, and SU. NM performed manuscript writing and data analysis. KF performed manuscript editing and data analysis. YN, TM, MNo, HT, AE, AH, KY, and HU supervised the work. All authors contributed to the article and approved the submitted version.

## Conflict of Interest

The authors declare that the research was conducted in the absence of any commercial or financial relationships that could be construed as a potential conflict of interest.

## Publisher’s Note

All claims expressed in this article are solely those of the authors and do not necessarily represent those of their affiliated organizations, or those of the publisher, the editors and the reviewers. Any product that may be evaluated in this article, or claim that may be made by its manufacturer, is not guaranteed or endorsed by the publisher.
