# Supplementary material for: Comparison of Abiraterone and Combined Androgen Blockade Therapy for High-Risk Metastatic Hormone-Sensitive Prostate Cancer: A Propensity Score-Matched Analysis
Source: Front Oncol. 2021 Dec 21;11:769068. doi: 10.3389/fonc.2021.769068 (PMC8724311; doi:10.3389/fonc.2021.769068)
Supplement: Supplementary file 1 [file Table_1.pdf]

Supplementary Table S1 Secondary therapy for mHSPC patients who have progressed to mCRPC

	AAP+ADT (n=11)	CAB (n=61)
Summary of secondary therapy, n (%)		
Flutamide	0 (0)	25 (41.0)
Enzalutamide	7 (58.3)	8 (13.1)
Abiraterone	0 (0)	12 (19.7)
Apalutamide	0 (0)	1 (1.6)
Docetaxel	3 (25.0)	11 (18.0)
Dexamethasone	1 (8.3)	3 (4.9)
Etinolestridol	0 (0)	1 (1.6)

Abbreviations: mHSPC, metastatic hormone-sensitive prostate cancer; mCRPC, metastatic castration-resistant prostate cancer; AAP, abiraterone acetate plus prednisone; ADT, androgen deprivation therapy; CAB, combined androgen blockade
